# The Chain Mediation Role of Self-Efficacy, Health Literacy, and Physical Exercise in the Relationship Between Internet Use and Older Adults’ Health: Cross-Sectional Questionnaire Study

**DOI:** 10.2196/73242

**Published:** 2025-07-01

**Authors:** Weizhen Liao, Chengyu Ma, Xiqiao Liu, Ziwei Sun

**Affiliations:** 1 School of Public Health Capital Medical University Beijing China

**Keywords:** chain mediation effect, digital engagement, older adult population, subjective health status

## Abstract

**Background:**

With the widespread use of the internet, the number of older internet users is rapidly increasing. However, the role of individual factors such as self-efficacy, health literacy, and physical exercise in the chain of influence between internet use and the health of older adults is unclear.

**Objective:**

On the basis of the media effects theory and the health belief model, we aimed to explore the relationship between internet use and self-rated health among Chinese older adults. We also analyzed the mediating roles of self-efficacy, health literacy, and physical exercise and examined how these mediating effects varied by sex, residence, and per capita monthly household income groups. In addition, we investigated the moderating effect of education level.

**Methods:**

We included 1147 participants aged ≥60 years from the 2021 Psychology and Behavior Investigation of Chinese Residents project in this study. Using a combination of multiple linear regression and bootstrap testing, we constructed a chain mediation model and a moderated chain mediation model to examine how internet use affects older adults’ health through self-efficacy, health literacy, and physical exercise. In addition, we explored the marginal effects of education level within each mediation pathway.

**Results:**

Internet use significantly improved the self-rated health of older adults (B=2.183; *P*<.001), and this improvement was exclusively mediated by self-efficacy (B=0.502; *P*<.001), health literacy (B=5.415; *P*<.001), and physical exercise (B=3.449; *P*<.001). These three factors could act as independent mediators or form sequential chain mediation pathways. In heterogeneity analysis, the total indirect effects were more pronounced among female participants (B=1.965; *P*<.05) and individuals with middle (B=1.971; *P*<.05) to high (B=2.710; *P*<.05) income levels. Furthermore, education level moderated the relationships between internet use and self-efficacy (B=0.452; *P*=.003) and between internet use and self-rated health (B=1.284; *P*=.01). This suggests that the positive influence of internet use on self-rated health was more pronounced among older adults with higher education.

**Conclusions:**

The findings of this study suggested that internet use can positively influence older adults’ self-rated health through the chain mediated effects of self-efficacy, health literacy, and physical exercise. This chain mediated effect was more pronounced among those with higher levels of education. In the future, efforts should be made to promote internet use among older persons by developing age-friendly digital platforms and expanding digital training and health education. Moreover, older persons should be encouraged to participate in volunteer activities to increase their self-efficacy and improve their health.

## Introduction

### Background

The wave of population aging is sweeping across the globe, and China, the second most populous country, is also experiencing an accelerated aging process. By the end of 2023, the population of individuals aged ≥60 years in China reached 296.97 million, accounting for 21.1% of the total population [1]. Meanwhile, along with the development and popularization of the internet, the number of older internet users is growing. As of December 2023, the number of internet users in China aged ≥60 years reached 170 million, accounting for 15.6% of the country’s internet users, and this share is still increasing [2].

The internet is playing an increasingly important role in the daily lives of older adults. Older adults can use the internet to contact friends and relatives, read news, and stay in touch with society. In addition, they can use the internet to watch videos, access health-related information, and engage in online shopping to facilitate their lives. Therefore, scholars have shown keen interest in understanding how internet use affects the health of older adults. Most studies have found positive effects of internet use on the health of older adults, such as reduced loneliness and depression [3], increased well-being [4], enhanced cognitive functioning [5], and better chronic disease management [6]. Internet use has also been associated with healthy behaviors, including physical exercise and preventive health actions [7,8]. However, some studies suggest that excessive internet use may have negative effects on older adults, such as reducing real-life social interactions [9] and lowering life satisfaction [10].

Beyond these general associations, researchers have explored the underlying mechanisms through which internet use affects health. Most existing studies have examined, from the perspective of social participation, the pathways through which social capital influences the relationship between internet use and health among older adults, focusing on factors such as social engagement, social trust, and social support [11-13]. In contrast, less attention has been paid to the individual-level psychological and behavioral mechanisms through which internet use affects health. Although studies have pointed out the positive effects of internet use on older adults’ self-efficacy [14], health literacy [14], and lifestyle behaviors [15], few studies have systematically examined how these factors interact within a chain mediation framework. Moreover, the moderating role of education in shaping these mechanisms has received limited attention in existing research, despite its potential importance in understanding disparities in health outcomes.

Addressing these gaps, we aimed to investigate the association between internet use and health among older adults in China, focusing on the chain mediation roles of self-efficacy, health literacy, and physical exercise. We also examined whether education level moderates these mediation paths. Drawing on nationally representative data from the 2021 Psychology and Behavior Investigation of Chinese Residents (PBICR) project, this study contributes to a more comprehensive understanding of how digital engagement supports healthy aging and offers policy implications at both the individual and societal levels.

### Theoretical Foundation

#### Media Effects Theory

Media effects theory explores how media use affects the cognition, emotions, attitudes, and behaviors of individuals or groups [16,17]. The theory posits that such influences are often indirect, meaning that media use first affects psychological factors such as attitudes and beliefs, which in turn shape behavior [16]. In the digital age, the internet has become an important medium through which older adults learn new skills, maintain social connections, and access health-related information. These experiences help enhance their self-efficacy [18] and improve their health knowledge [19]. Moreover, internet use can improve older adults’ health literacy [20], enabling them to better understand and apply health information, thereby facilitating the adoption of health-promoting behaviors [21].

#### Health Belief Model

The health belief model (HBM) is a sociopsychological model developed by social psychologists in the US Public Health Service in the 1950s to explain and predict individuals’ health-related behaviors [22]. It has been widely used in the field of digital health. For example, researchers have used the HBM to examine factors influencing women’s adoption of diet and exercise content on social media during the COVID-19 pandemic [23] and to analyze determinants of menopausal women’s intention to use mental health applications [24]. In addition, the HBM has been used to investigate the role of social media in promoting public health behaviors [25]. In the HBM, when individuals possess a high level of self-efficacy, they are more confident in their ability to successfully execute health behaviors and achieve healthy outcomes. Consequently, they are more likely to overcome obstacles to behavior change and adopt healthy behaviors. For instance, studies have shown that improving students’ self-efficacy can lead to better oral hygiene behaviors and improved oral health outcomes [26]. Furthermore, self-efficacy has been identified as a significant predictor of older adults’ online health information–seeking behavior [27], further supporting its crucial role in facilitating health behavior.

**Figure 1:**
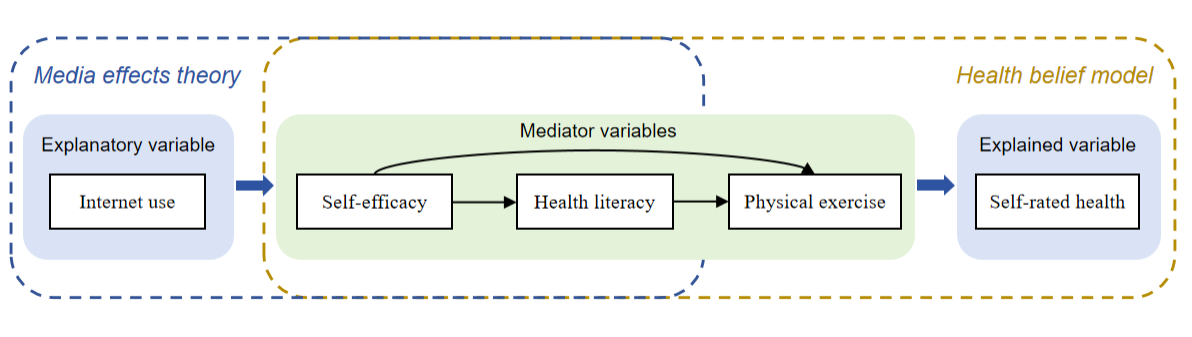
Chain mediation pathway of self-efficacy, health literacy, and physical exercise.

In summary, the media effects theory explains how internet use can influence individuals’ attitudes (such as self-efficacy) and their ability to internalize health information (health literacy). The HBM illustrates how self-efficacy drives the transformation of these cognitive factors into health behaviors (such as physical exercise). By integrating the media effects theory and the HBM, this study constructs a logical pathway from media use to cognitive and attitudinal changes, ultimately leading to health behaviors and outcomes. This framework explains how internet use indirectly influences self-rated health through the mediating roles of self-efficacy, health literacy, and physical exercise (Figure 1).

### Research Hypotheses

#### Relationship Between Internet Use and Older Adults’ Health

Existing research has revealed that internet use among older adults impacts their self-rated health, mental health, physical health, and chronic disease management. Internet use can improve cognitive function by increasing activity participation [28], and it has also been shown to positively influence objective physical health [29]. In chronic disease management, internet use effectively increases physical exercise, aiding in the prevention of obesity, heart disease, hypertension, diabetes, and other chronic conditions [30]. Among various health indicators, self-rated health is a comprehensive measure and a powerful predictor of well-being [31]. Research has shown that internet use may enhance self-rated health. For instance, Zhou et al [32] found a positive correlation between internet use and self-rated health; Liu et al [13] discovered that Chinese older adult internet users reported better health. Therefore, we focused on self-rated health and proposed the following hypothesis:

- Hypothesis 1: internet use positively influences the self-rated health among older adults.

#### Mediating Role of Self-Efficacy

Self-efficacy refers to an individual’s perceived ability and confidence to handle challenges in different situations, and it is considered a relatively stable psychological trait [33]. Previous research has identified a significant positive correlation between internet use and self-efficacy in older adults [34]. The use of information and communication technology allows older adults to learn new skills and remain socially active, which helps to increase their self-efficacy and reduce their social isolation [18]. Furthermore, according to the HBM, self-efficacy can promote health-related behaviors. For example, one study [27] found that higher self-efficacy can positively influence health information–seeking intentions by enhancing expectations of health-related outcomes, thus encouraging positive health management behaviors. To summarize, these findings suggest that internet use may improve older adults’ health by enhancing their self-efficacy. Therefore, the following research hypothesis was proposed:

- Hypothesis 2: self-efficacy is a mediator between internet use and self-rated health among older adults.

#### Mediating Role of Health Literacy

Health literacy is defined as the ability to acquire, process, understand, and communicate information related to health. This ability facilitates healthy decision-making and the management of health conditions [35]. Higher levels of health literacy are advantageous for both physical and mental well-being [36,37]. Internet use has the potential to elevate health literacy by enhancing access to health information [38]. It provides older adults with convenient channels to obtain scientific and authoritative health knowledge and information. This information not only boosts their health literacy but also guides them to adopt healthier lifestyles, such as balanced diets and moderate exercise, thereby promoting their overall well-being. Moreover, individuals with higher health literacy are more likely to understand and adhere to medical advice and protocols, leading to improved health outcomes [39]. Studies have found a significant correlation between frequent internet use, good health status, and higher health literacy [40]. Therefore, the following research hypothesis was proposed:

- Hypothesis 3: health literacy is a mediator between internet use and self-rated health among older adults.

#### Mediating Role of Physical Exercise

Health behaviors typically refer to a series of positive lifestyle choices aimed at disease prevention and promotion of physical and mental well-being. Physical exercise is a crucial determinant of health, capable of preventing numerous diseases, including cardiovascular diseases [41]. Previous studies have indicated a link between high levels of internet use and high levels of physical exercise [42]. Guo et al [15], using data from the 2018 China Health and Retirement Longitudinal Study, found that internet use and its frequency significantly increased the probability and frequency of physical exercise participation among middle-aged and older adults. Physical exercise not only benefits physical health but also positively influences emotional states such as anxiety, stress, and depression through physiological and biochemical mechanisms, including the promotion of dopamine secretion [43], thereby contributing to mental well-being. Zhang and Zhang [44] pointed out that the internet facilitates physical exercise in multiple ways, and active exercise further impacts individuals’ mental health status. Liu et al [45] found that longer internet use predicted lower levels of depressive symptoms, and the frequency of physical activities, such as exercise, mediated the longitudinal relationship between internet use and depressive symptoms among older adults. Therefore, the fourth research hypothesis of this paper was proposed as follows:

- Hypothesis 4: physical exercise is a mediator between internet use and self-rated health among older adults.

#### Chain Mediating Effects of Self-Efficacy, Health Literacy, and Physical Exercise

The effects of self-efficacy, health literacy, and physical activity on health do not exist independently but may interact to form a chain mediation pathway linking internet use and health.

First, self-efficacy has been shown to positively influence health literacy. Individuals with high self-efficacy tend to be more proactive in acquiring health-related knowledge and are more motivated to adopt healthy socialization and lifestyles [46,47].

Second, self-efficacy is a strong predictor of physical exercise [48,49]. Older adults with high self-efficacy are more confident in achieving health goals, which motivates them to initiate and maintain physical exercise routines [50].

Third, health literacy positively influences physical exercise [51,52]. Individuals with higher health literacy are better able to understand and act upon health information [53], which helps increase their participation in physical exercise [54]. High health literacy enables older adults to accurately assess their health status, adopt health advice, and serve as a basis for physical exercise.

These variables are not only interrelated but also follow a progressive pathway. Internet use may enhance older adults’ self-efficacy, which subsequently improves their health literacy, promotes engagement in physical exercise, and ultimately contributes to better health outcomes. Therefore, the following research hypotheses were proposed:

- Hypothesis 5: self-efficacy and health literacy form a chain mediation pathway between internet use and self-rated health among older adults.
- Hypothesis 6: self-efficacy and physical exercise form a chain mediation pathway between internet use and self-rated health among older adults.
- Hypothesis 7: health literacy and physical exercise form a chain mediation pathway between internet use and self-rated health among older adults.
- Hypothesis 8: self-efficacy, health literacy, and physical exercise form a chain mediation pathway between internet use and self-rated health among older adults.

#### Moderating Effect of Education Level

Previous studies have found that education level is a significant factor influencing internet use among older adults. Education levels reflect disparities in cultural capital [55], which may influence the relationship between internet use and health among older adults [56]. Compared to their less-educated counterparts, older adults with higher education levels have greater access to the internet and are more likely to acquire, understand, and learn health-related information online. Therefore, we argue that education level moderates the indirect pathways linking internet use to health. In other words, different levels of education may alter the strength of the mediation effects between internet use and self-rated health. On this basis, the following hypothesis was proposed:

- Hypothesis 9: education level positively moderates the relationship between internet use and self-rated health among older adults.

**Figure 2:**
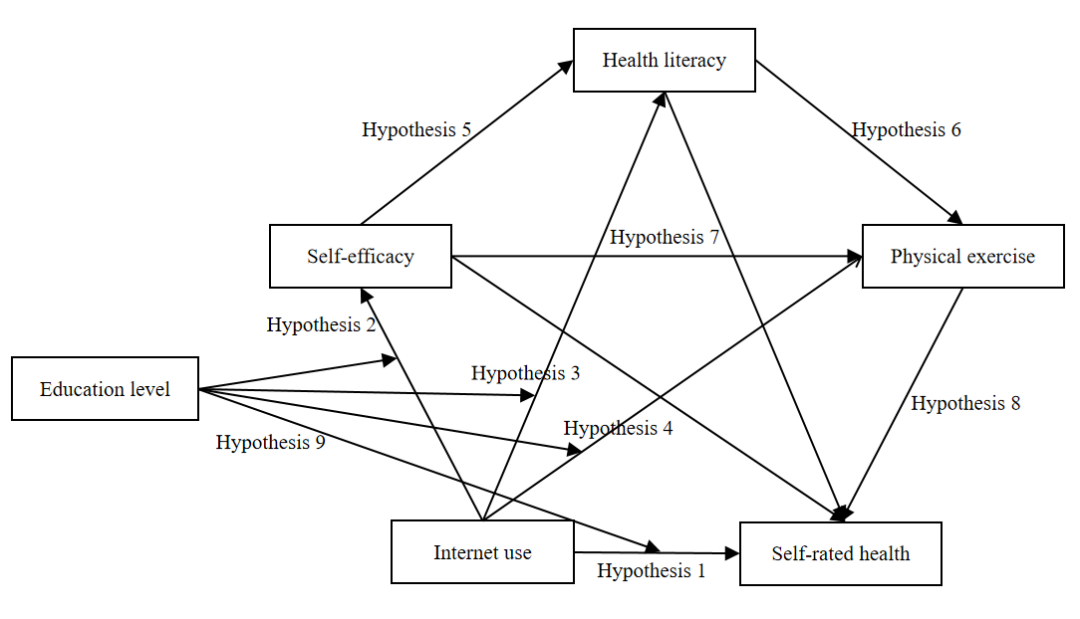
Hypothesis model. Hypothesis 1: internet use to self-rated health; hypothesis 2: internet use to self-efficacy to self-rated health; hypothesis 3: internet use to health literacy to self-rated health; hypothesis 4: internet use to physical exercise to self-rated health; hypothesis 5: internet use to self-efficacy to health literacy to self-rated health; hypothesis 6: internet use to self-efficacy to physical exercise to self-rated health; hypothesis 7: internet use to health literacy to physical exercise to self-rated health; hypothesis 8: internet use to self-efficacy to health literacy to physical exercise to self-rated health; hypothesis 9: adjustment of education level—internet use to self-rated health.

The theoretical hypothesis model, on the basis of the aforementioned research hypotheses, is shown in Figure 2.

## Methods

### Data Source

The data used in this study are the results of the 2021 survey of the PBICR project (the complete questionnaire used in the study can be found in Multimedia Appendix 1). Organized by the School of Public Health at Peking University, the PBICR project has conducted annual surveys since 2020, collecting data across 4 waves. The most recent publicly available data are from 2021. The project used a multistage sampling method to select 120 cities from 23 provinces, 5 autonomous regions, and 4 municipalities in China. All provincial capitals and municipalities were included by default. In addition, 2 to 6 noncapital prefecture-level cities in each province or region were selected using a random number table, ensuring randomization and regional diversity (detailed information on the survey regions can be found in Multimedia Appendix 2).

Within each selected city, quota sampling was used based on the 2021 national census data, matching local distributions in age, sex, and urban-rural residency. A total of 11,032 valid responses were collected. For this study, we focused on respondents aged ≥60 years, resulting in a final sample of 1147 older adults.

### Ethical Considerations

This study was conducted in accordance with the ethical principles outlined in the Declaration of Helsinki and the International Ethical Guidelines for Biomedical Research Involving Human Subjects. Ethical approval was obtained from the Ethics Committee of the Shaanxi Provincial Research Center for Health Culture (approval JKWH-2021-01) and the Ethics Committee of Jinan University (approval JNUKY-2021-018). All participants provided written informed consent prior to participation. No compensation was provided to participants in this study. The research protocol was designed to ensure the protection of participants’ privacy and the confidentiality of the collected data.

### Variable Setting

#### Explained Variable

Self-rated health was chosen as the explained variable. The survey question used to assess self-rated health was, “How would you rate your health today?” Respondents were asked to rate their health on a scale from 0 to 100, with higher scores indicating better self-rated health.

#### Explanatory Variable

The explanatory variable in this study was internet use. The relevant question in the survey was, “How often do you typically use the following media?” Internet use was measured based on the reported frequency of using PCs (including tablets) or smartphones. Participants were categorized into a 5-category ordinal variable according to their use frequency. Detailed variable coding is presented in Table 1.

**Table 1:** Definition of each variable.

Variable types and variable names			Variable definition
**Explained variable**			
	Self-rated health	Self-rated health score ranged from 0 to 100	
**Explanatory variable**			
	Internet use	Never=1; occasionally (≤1 d per wk)=2; sometimes (2-3 d per wk)=3; frequently (4-5 d per wk)=4; almost every day (6-7 d per wk)=5	
**Control variables**			
	Age group (y)	60-69=1; 70-79=2; ≥80=3	
	Sex	Male=1; female=0	
	Education level	Junior high school and below=1; high school or vocational school=2; university (including junior college) and above=3	
	Marital status	Married=1; other (including divorced, unmarried, and widowed)=0	
	Residence	Urban=1; rural=0	
	Per capita monthly household income (RMB)^a^	≤3000=1; 3001-6000=2; ＞6000=3^a^	
	Living situation	Living alone=1; other=0	
	Medical insurance	Participate in medical insurance (including urban employee medical insurance, urban and rural resident medical insurance, commercial medical insurance, and public medical insurance)=1; out-of-pocket spending=0	
	Chronic diseases (n)	Experiencing 1 or no chronic disease=1; experiencing 2 chronic diseases=2; experiencing 3 chronic diseases=3; experiencing 4 or more chronic diseases=4	
**Mediator variables**			
	Self-efficacy	Self-efficacy score ranged from 8 to 40	
	Health literacy	Inadequate=1; problematic=2; sufficient=3; excellent=4	
	Physical exercise	0 min=1; within 150 min=2; >150 min=3	

^a^RMB ¥1=US $0.155 (2021 average).

#### Control Variables

The selection of control variables was informed by previous studies on the association between internet use and health in older adults [8,12,13]. These included individual characteristics (age, sex, and education level); family-related factors (marital status and living situation); and socioeconomic conditions (residence, per capita monthly household income, and medical insurance). In addition, the number of chronic diseases experienced was included as a key health-related covariate that may influence self-rated health. These variables were included to reduce potential confounding and improve the robustness of the estimated mediation effects. The specific settings for each variable are presented in Table 1.

#### Mediator Variables

The mediator variables selected for this study included self-efficacy, health literacy, and physical exercise (Table 1).

Self-efficacy was measured using the New General Self-Efficacy Scale, developed by Chen et al [57] in 2001. It consists of 8 items, with responses ranging from 1 (strongly disagree) to 5 (strongly agree). The total score ranges from 8 to 40, with higher scores reflecting greater levels of general self-efficacy.

Health literacy was assessed using the 12-item Short-Form Health Literacy Questionnaire [58], which consists of 3 assessment dimensions: health care, disease prevention, and health promotion. Each item is scored on a 4-point Likert scale (1=very difficult; 2=difficult; 3=easy; and 4=very easy). The health literacy index was standardized using the following formula: [index = (mean – 1) × (50/3)], where the “mean” represents the average score for each individual. The resulting health literacy scores ranged from 0 to 50, with scores of 0 to 25 classified as inadequate, 26 to 33 as problematic, 34 to 42 as sufficient, and 43 to 50 as excellent [59].

Physical exercise was measured based on the weekly total exercise time. Activities included fitness exercises (eg, dumbbell lifting), walking, swimming, cycling, using sports equipment (eg, treadmills), and other forms of aerobic exercise (eg, running and table tennis). The total exercise time (in minutes) was calculated by summing the duration of all activities, with ≥150 minutes per week considered a healthy level of physical exercise [60].

#### Moderating Variable

The moderating variable selected for this study was the education level of older adults. Education level was assessed using the questionnaire item, “Your highest level of education,” with responses categorized into the following groups: junior high school or below, high school or junior college, and bachelor’s degree or higher. These categories were determined based on the characteristics of the sample population and the available data.

### Statistical Methods

The statistical analysis of this study was conducted in 5 main stages. First, a descriptive statistical analysis was performed. Second, a multiple linear regression model was used to preliminarily examine the relationship between internet use and self-rated health. Third, a chain mediation analysis was conducted to test whether self-efficacy, health literacy, and physical exercise sequentially mediated the relationship between internet use and self-rated health. Fourth, heterogeneity analyses were performed to examine whether the mediation effects varied by sex, residence, and per capita monthly household income groups. Fifth, a moderated mediation analysis was conducted to assess whether education level moderated the specific mediation paths within the chain model.

In addition, to assess the validity of self-rated health as an explained variable, an additional analysis was conducted to examine its association with BMI. This analysis was intended to examine whether subjective health perceptions aligned with objective health indicator.

All mediation and moderation analyses were conducted using the PROCESS macro (version 4.0), developed by Hayes and Rockwood [61], with 5000 bootstrap resamples. A mediation or moderation effect was considered statistically significant if the 95% CI did not include 0. All statistical analyses were performed using SPSS (version 25.0; IBM Corp), with 2-sided tests and a significance level set at *P*<.05.

## Results

### Descriptive Analysis

Participant characteristics are presented in Table 2. The mean self-rated health score of the 1147 participants was 73.90 (SD 19.61). Among them, 356 (31.04%) participants used the internet almost daily. Most of the participants (525/1147, 45.77%) were aged between 70 and 79 years. Sex distribution was approximately equal (581/1147, 50.65% men and 566/1147, 49.35% women). Most older adults had a junior high school education or less (737/1147, 64.25%), and most were married (894/1147, 77.94%).

**Table 2:** Descriptive statistical results of each variable (N=1147).

Variable types and variable names					Values
**Explained variable**					
	Self-rated health, mean (SD)			73.90 (19.61)	
**Explanatory variable**					
	**Internet use, n (%)**				
		Never	317 (27.64)		
		Occasionally	97 (8.46)		
		Sometimes	183 (15.95)		
		Frequently	194 (16.91)		
		Almost every day	356 (31.04)		
**Control variables**					
	**Age group (y), n (%)**				
		60-69	514 (44.81)		
		70-79	525 (45.77)		
		≥80	108 (9.42)		
	**Sex** **, n (%)**				
		Male	581 (50.65)		
		Female	566 (49.35)		
	**Education level, n (%)**				
		Junior high school and below	737 (64.25)		
		High school or vocational school	200 (17.44)		
		University and above	210 (18.31)		
	**Marital status, n (%)**				
		Married	894 (77.94)		
		Other	253 (22.06)		
	**Residence, n (%)**				
		Urban	654 (57.02)		
		Rural	493 (42.98)		
	**Per capita monthly household income (RMB)^a^, n (%)**				
		≤3000	471 (41.06)		
		3001-6000	406 (35.4)		
		＞6000	270 (23.54)		
	**Living situation, n (%)**				
		Living alone	136 (11.86)		
		Other	1011 (88.14)		
	**Medical insurance, n (%)**				
		Participate in medical insurance	1017 (88.67)		
		Out-of-pocket spending	130 (11.33)		
	**Chronic diseases (n), n (%)**				
		≤1	831 (72.45)		
		2	207 (18.05)		
		3	74 (6.45)		
		>4	35 (3.05)		
**Mediator variables**					
	Self-efficacy, mean (SD)			28.10 (5.34)	
	**Health literacy, n (%)**				
		Inadequate	339 (29.56)		
		Problematic	574 (50.04)		
		Sufficient	146 (12.73)		
		Excellent	88 (7.67)		
	**Physical exercise, n (%)**				
		0 min	121 (10.55)		
		<150 min	362 (31.56)		
		>150 min	664 (57.89)		

^a^RMB ¥1=US $0.155 (2021 average).

There were more urban residents (654/1147, 57.02%) than rural residents (493/1147, 42.98%). The average monthly per capita household income primarily fell into 2 categories: ≤RMB 3000 (US $465) or less (471/1147, 41.06%) and RMB 3001 (US $465.15) to RMB 6000 (US $930; 406/1147, 35.4%). Most participants did not live alone (1011/1147, 88.14%), and most participants had health insurance (1017/1147, 88.67%). In addition, 831 participants (72.45%) had at most one chronic medical condition.

The mean self-efficacy score for participants was 28.10 (SD 5.34). In terms of health literacy, most participants were classified as inadequate (339/1147, 29.56%) or problematic (574/1147, 50.04%). More than half of the older adults (664/1147, 57.89%) were engaged in at least 150 minutes of physical exercise per week.

### The Relationship Between Internet Use and Self-Rated Health

Multiple linear regression analyses were conducted with self-rated health as the explained variable and internet use as the explanatory variable. Collinearity was also assessed. The results of the analyses are shown in Table 3. The results of collinearity diagnosis showed that the variance inflation factor values for all variables in both models A and B were <10, indicating the absence of significant multicollinearity.

**Table 3:** Results of multiple linear regression analysis.

Variables				Model A^a^ (self-rated health)										Model B^b^ (self-rated health)								
				B^c^			*P* value			VIF^d^			B				*P* value			VIF		
**Explanatory variable**																						
	Internet use	2.182			<.001			1.269			0.474				.23			1.480				
**Control variables**																						
	Age	−0.180			.84			1.093			−0.405				.63			1.097				
	Sex	1.075			.33			1.036			−0.099				.93			1.059				
	Education level	2.056			.01			1.404			0.976				.22			1.428				
	Marital status	3.399			.03			1.421			2.696				.07			1.425				
	Residence	0.871			.48			1.222			−0.641				.58			1.247				
	Per capita monthly household income	−0.409			.62			1.376			−0.653				.40			1.386				
	Living situation	4.694			.02			1.368			4.575				.01			1.370				
	Medical insurance	6.343			<.001			1.024			3.924				.02			1.045				
	Chronic diseases	−5.275			<.001			1.040			−4.584				<.001			1.053				
**Mediator variables**																						
	Self-efficacy	N/A^e^			N/A			N/A			0.502				<.001			1.221				
	Health literacy	N/A			N/A			N/A			5.415				<.001			1.368				
	Physical exercise	N/A			N/A			N/A			3.449				<.001			1.268				
Constant			62.414			<.001			N/A			40.091				<.001			N/A			

^a^*R*^2^=0.116; *F*_10, 1136_=14.911; *P<*.001.

^b^*R*^2^=0.212; *F*_13, 1133_=23.461; *P<*.001.

^c^B: unstandardized regression coefficient.

^d^VIF: variance inflation factor.

^e^N/A: not applicable.

Compared with model A, model B had 3 new control variables: *self-efficacy*, *health literacy*, and *exercise level*. As shown in model A, internet use had a significant positive effect on self-rated health in older adults (B=2.182, *P*<.001), suggesting that internet use may enhance the self-rated health of older adults. Model B further showed that *self-efficacy*, *health literacy*, and *physical exercise* could positively influence older adults’ self-rated health (*P*<.001). Comparing models A and B, we found that, after adding the 3 variables of *self-efficacy*, *health literacy*, and *physical* *exercise*, the significance of the regression coefficients of internet use on older adults’ self-rated health disappeared (B=0.474; *P*=.23). This finding suggests that *self-efficacy*, *health literacy*, and *physical exercise* may mediate the relationship between internet use and older adults’ self-rated health.

### Chain Mediation Analysis

To explore the roles of *self-efficacy*, *health literacy*, and *physical exercise* in the relationship between internet use and self-rated health among older adults, we further conducted a chain mediation analysis [61]. In the chain mediation analysis, *self-efficacy* was a continuous variable, and all other variables were categorical. Therefore, *self-efficacy* scores were standardized before analysis.

The results of the chain mediation analysis for internet use and self-rated health are presented in Table 4. According to model 1, internet use significantly predicted self-rated health (B=2.183; *P*<.001), supporting hypothesis 1 and suggesting that internet use had a positive impact on older adults’ self-rated health. Models 2, 3, and 4 showed that internet use significantly predicted *self-efficacy* (B=0.868, *P*<.001); *health literacy* (B=0.130, *P*<.001); and *physical exercise* (B=0.088, *P*<.001).

**Table 4:** Regression results of the relationship among internet use, self-efficacy, health literacy, physical exercise, and self-rated health.

Variables			Model 1^a^ (self-rated health)					Model 2^b^ (self-efficacy)					Model 3^c^ (health literacy)					Model 4^d^ (physical exercise)					Model 5^e^ (self-rated health)		
			B^f^		*P* value		B			*P* value		B			*P* value		B			*P* value		B			*P* value
**Explanatory variable**																									
	Internet use	2.183		<.001		0.868			<.001		0.13			<.001		0.088			<.001		0.474			.23	
**Mediator variables**																									
	Self-efficacy	N/A^g^		N/A		N/A			N/A		0.046			<.001		0.005			.21		0.502			<.001	
	Health literacy	N/A		N/A		N/A			N/A		N/A			N/A		0.068			.006		5.415			<.001	
	Physical exercise	N/A		N/A		N/A			N/A		N/A			N/A		N/A			N/A		3.449			<.001	
**Control variables**																									
	Age (y)	−0.180		.84		0.469			.05		−0.013			.71		−0.019			.51		−0.405			.63	
	Sex	1.075		.33		0.400			.19		0.048			.27		0.171			<.001		−0.099			.93	
	Education level	2.056		.01		0.409			.07		0.084			.01		0.084			.002		0.976			.22	
	Marital status	3.400		.03		0.441			.31		0.079			.20		−0.025			.62		2.696			.07	
	Residence	0.871		.48		0.270			.42		0.141			.004		0.148			<.001		−0.641			.58	
	Per capita monthly household income	−0.410		.62		−0.179			.43		0.022			.50		0.075			.005		−0.653			.40	
	Living situation	4.694		.02		−0.494			.36		0.047			.55		0.069			.29		4.575			.01	
	Medical insurance	6.343		<.001		1.409			.003		0.118			.09		0.191			.001		3.924			.02	
	Chronic diseases	−5.275		<.001		0.019			.93		−0.096			.001		−0.047			.06		−4.584			<.001	
Constant			62.414		<.001		−5.728			<.001		1.287			<.001		1.565			<.001		54.193			<.001

^a^*R*^2^=0.116; *F*_10, 1136_=14.911; *P*<.001.

^b^*R*^2^=0.098; *F*_10, 1136_=12.328; *P*<.001.

^c^*R*^2^=0.264; *F*_11, 1135_=37.055; *P*<.001.

^d^*R*^2^=0.211; *F*_12, 1134_=25.297; *P*<.001.

^e^*R*^2^=0.212; *F*_13, 1133_=23.461; *P*<.001.

^f^B: unstandardized regression coefficient.

^g^N/A: not applicable.

When internet use, *self-efficacy*, *health literacy*, and *physical exercise* were all included in the regression equation (model 5), the coefficients of the variables changed. Compared to model 1, the direct effect of internet use on self-rated health became nonsignificant in model 5 (B=0.474; *P*=.23). In contrast, *self-efficacy*, *health literacy*, and *physical exercise* remained significant predictors (all *P*<.001), which is consistent with the results of the multiple linear regression analysis.

**Figure 3:**
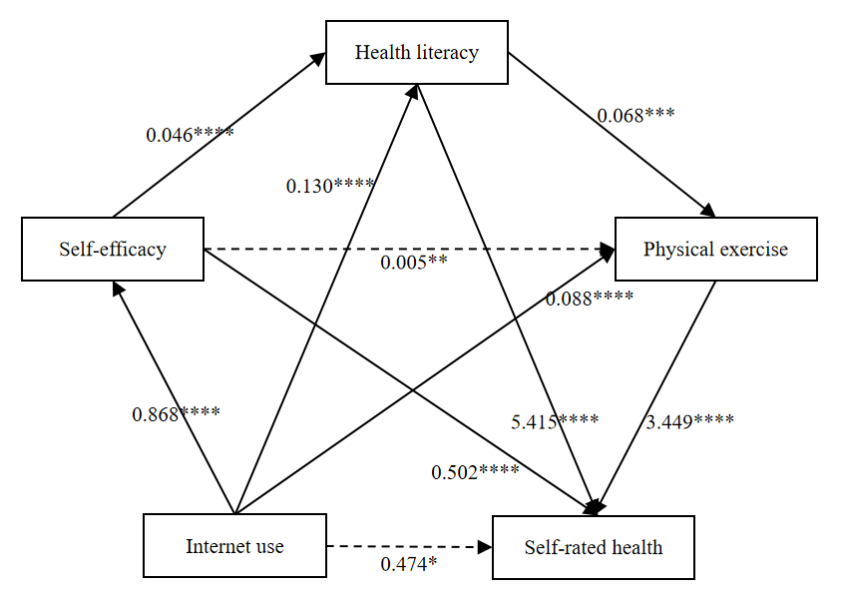
Chain mediation analysis of the association between internet use and self-rated health. ****P<.001; ***P=.006; **P=.21; *P=.23.

Figure 3 and Table 5 together illustrate the results of the chain mediation analysis. In Figure 3, solid arrows indicate statistically significant paths, while dashed arrows represent nonsignificant relationships. The corresponding bootstrap test results are reported in Table 5.

**Table 5:** Results of the chain mediation analysis.

Effect path	B^a^ (SE; 95% CI)	Proportion of effect (%)
Total effect	2.183 (0.384;1.429 to 2.936)	N/A^b^
Direct effect	0.474 (0.392; −0.295 to 1.243)	N/A
Total indirect effect	1.709 (0.203; 1.325 to 2.129)	100
Internet use→self-efficacy→self-rated health	0.435 (0.124; 0.215 to 0.699)	25.48
Internet use→health literacy→self-rated health	0.703 (0.123; 0.470 to 0.957)	41.11
Internet use→physical exercise→self-rated health	0.303 (0.094; 0.136 to 0.496)	17.71
Internet use→self-efficacy→health literacy→self-rated health	0.215 (0.042; 0.139 to 0.301)	12.56
Internet use→self-efficacy→physical exercise→self-rated health	0.014 (0.013; −0.010 to 0.042)	0.81
Internet use→health literacy→physical exercise→self-rated health	0.030 (0.015; 0.007 to 0.065)	1.77
Internet use→self-efficacy→health literacy→physical exercise→self-rated health	0.009 (0.005; 0.002 to 0.020)	0.54

^a^B: unstandardized regression coefficient.

^b^N/A: not applicable.

As shown in Figure 3 and Table 5, the total effect of internet use on self-rated health was 2.183, with a 95% CI excluding 0, which was consistent with the result from model 1. This suggests that internet use had a positive impact on older adults’ self-rated health. The direct effect was 0.474, and its 95% CI included 0. This suggests that after controlling for the 3 mediating variables, internet use itself did not have a significant direct effect on older adults’ self-rated health. Instead, its positive influence operated entirely through the mediating roles of *self-efficacy*, *health literacy*, and *physical exercise*. The total indirect effect was 1.709, suggesting that these three factors may have a positive overall mediating effect on the relationship between internet use and self-rated health.

According to the study hypotheses, there were 7 indirect paths in the chain mediation model, as shown in Table 5. The analysis results for paths 4 to 6 indicated the significant mediating effects of *self-efficacy*, *health literacy*, and *physical exercise*, thereby supporting hypotheses 2, 3, and 4. At the 95% CI, the mediation effects were 0.435, 0.703, and 0.303, accounting for 25.48%, 41.11%, and 17.71% of the total indirect effect, respectively. Among the chain mediation paths (paths 7 to 10), paths 7, 9, and 10 were statistically significant, while path 8 was not. Thus, hypothesis 6 was not supported, while hypotheses 5, 7, and 8 were confirmed.

### Heterogeneity Analysis

To explore whether the mediating effect of internet use on self-rated health varied by subgroup, we conducted heterogeneity analyses by sex, residence type, and per capita monthly household income. The results are shown in Table 6. In the total effect analysis, the positive association of internet use on self-rated health was statistically significant for all subgroups except for those with a per capita monthly household income ≤RMB 3000 (US $465; B=0.744; *P*>.05). In mediation analyses, the total indirect effect was consistently significant across groups, but this effect varied across mediation pathways. First, higher indirect effects and more effective mediation paths were observed in the female group than in the male group. Second, the effective mediating paths were generally consistent between rural and urban groups, suggesting that the ways in which internet use affected self-rated health were similar across these populations. Finally, the total indirect effect increased with monthly household income per capita, implying that the mediating effects of self-efficacy, health literacy, and physical exercise were more pronounced among the high-income group.

**Table 6:** Results of heterogeneity analysis.

Effect path	Sex		Residence		Per capita monthly household income (RMB)^a^		
	Female, B^b^	Male, B	Rural, B	Urban, B	≤3000, B	3001-6000, B	>6000, B
Total effect	2.391^c^	2.036^c^	2.012^c^	2.345^c^	0.744	2.605^c^	4.733^c^
Direct effect	0.426	0.563	0.388	0.648	−0.504	0.634	2.023
Total indirect effect	1.965^c^	1.473^c^	1.624^c^	1.697^c^	1.248^c^	1.971^c^	2.710^c^
Internet use→self-efficacy→self-rated health	0.695^c^	0.168	0.524^c^	0.348^c^	0.222	0.467^c^	0.804^c^
Internet use→health literacy→self-rated health	0.629^c^	0.776^c^	0.516^c^	0.840^c^	0.676^c^	0.840^c^	0.603^c^
Internet use→physical exercise→self-rated health	0.389^c^	0.212	0.267^c^	0.289^c^	0.068	0.448^c^	0.941^c^
Internet use→self-efficacy→health literacy→self-rated health	0.158^c^	0.290^c^	0.257^c^	0.179^c^	0.260^c^	0.158^c^	0.260^c^
Internet use→self-efficacy→physical exercise→self-rated health	0.000	0.021	0.028	0.004	0.012	−0.021	0.046
Internet use→health literacy→physical exercise→self-rated health	0.075^c^	0.004	0.021	0.031	0.008	0.065^c^	0.038
Internet use→self-efficacy→health literacy→physical exercise→self-rated health	0.019^c^	0.002	0.011	0.007	0.003	0.012^c^	0.017

^a^RMB ¥1=US $0.155 (2021 average).

^b^B: unstandardized regression coefficient.

^c^The CI did not include 0.

Notably, the mediation paths through “health literacy” and “self-efficacy→health literacy” were significant across all subgroups. These findings highlight that self-efficacy and health literacy play a key role in the association between internet use and self-rated health.

### Moderated Chain Mediation Analysis

The moderating effect of *education level* was tested, and the results are provided in Table 7. The results revealed that education level moderated the relationships between *internet use*, *self-efficacy*, and *self-rated health* (models 6 and 9). However, it did not moderate the relationships between *internet use*, *health literacy*, and *physical exercise* (models 7 and 8).

**Table 7:** Moderated chain mediation test of internet use on self-rated health.

Variables			Model 6^a^ (self-efficacy)					Model 7^b^ (health literacy)					Model 8^c^ (physical exercise)					Model 9^d^ (self-rated health)		
			B^e^		*P* value		B			*P* value		B			*P* value		B			*P* value
**Explanatory variable**																				
	Internet use	0.262		<.001		0.157			<.001		0.089			.001		−1.233			.12	
**Mediator variables**																				
	Self-efficacy	N/A^f^		N/A		0.046			<.001		0.005			.21		0.478			<.001	
	Health literacy	N/A		N/A		N/A			N/A		0.068			.006		5.463			<.001	
	Physical exercise	N/A		N/A		N/A			N/A		N/A			N/A		3.453			<.001	
**Moderating variables**																				
	Education level	−1.327		.03		0.163			.07		0.089			.24		−3.946			.07	
	Internet use×education level	0.452		.003		−0.021			.35		−0.001			.95		1.284			.01	
**Control variables**																				
	Age (y)	0.488		.04		−0.014			.69		−0.019			.51		−0.340			.69	
	Sex	0.456		.14		0.046			.30		0.171			<.001		0.066			.95	
	Marital status	0.419		.33		0.080			.20		−0.025			.62		2.641			.08	
	Residence	0.290		.39		0.140			.04		0.147			<.001		−0.586			.61	
	Per capita monthly household income	−0.137		.54		0.020			.54		0.075			.005		−0.540			.49	
	Living situation	−0.524		.33		0.048			.54		0.069			.29		4.475			.02	
	Medical insurance	1.398		.004		0.118			.09		0.191			.001		3.918			.02	
	Chronic diseases	0.028		.89		−0.097			.001		−0.047			.06		−4.554			<.001	
Constant			−3.674		.001		1.195			<.001		1.559			<.001		59.826			<.001

^a^*R*^2^=0.105; *F*_11, 1135_=12.096; *P*<.001.

^b^*R*^2^=0.265; *F*_12, 1134_=34.037; *P*<.001.

^c^*R*^2^=0.211; *F*_13, 1133_=23.331; *P*<.001.

^d^*R*^2^=0.216; *F*_14, 1132_=22.311; *P*<.001.

^e^B: unstandardized regression coefficient.

^f^N/A: not applicable.

**Figure 4:**
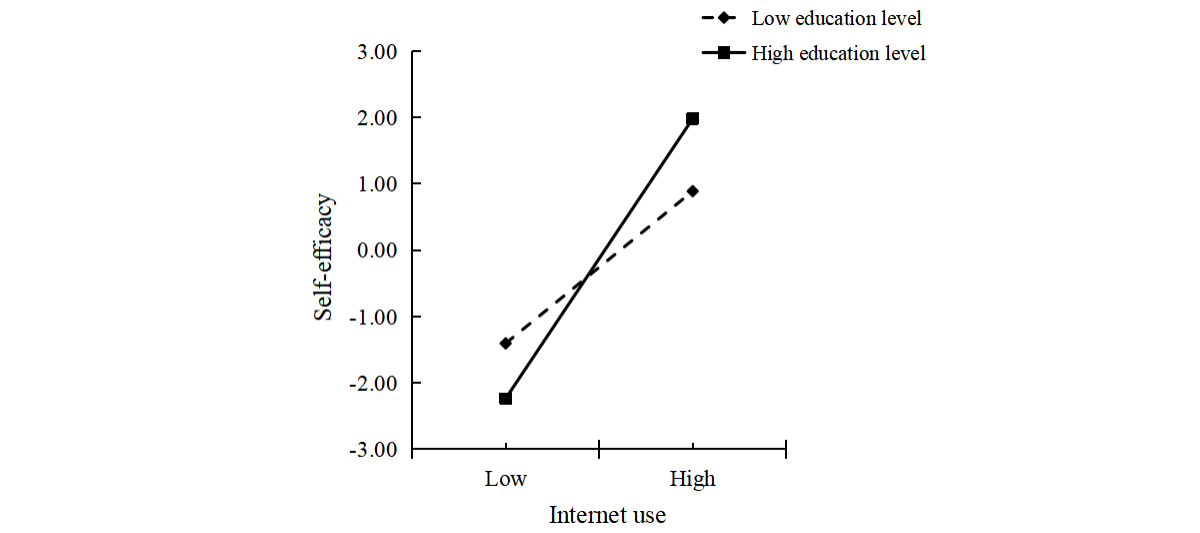
Education level as a moderator in the relationship between internet use and self-efficacy.

The interaction term between *internet use* and *education level* (B=0.452; *P*=.003) significantly predicted *self-efficacy*. Education level positively moderated the relationship between *internet use* and *self-efficacy*, as shown in Figure 4. At higher education levels, the effect of internet use on self-efficacy was stronger (B=1.313; *P*<.001), whereas at lower education levels, the effect was weaker (B=0.714; *P*<.001), as presented in Table 8. The slope for individuals with higher education was significantly steeper than that for those with lower education, indicating that among older adults with higher education, internet use had a more pronounced influence on *self-efficacy*.

**Table 8:** Moderating effect of education level.

Moderating variable	Education level	Self-efficacy			Self-rated health	
		Effect	*P* value	Effect		*P* value
Mean – SD	1.000	0.714	<.001	0.051		.90
Mean	1.541	0.959	<.001	0.745		.07
Mean + SD	2.325	1.313	<.001	1.752		.007

**Figure 5:**
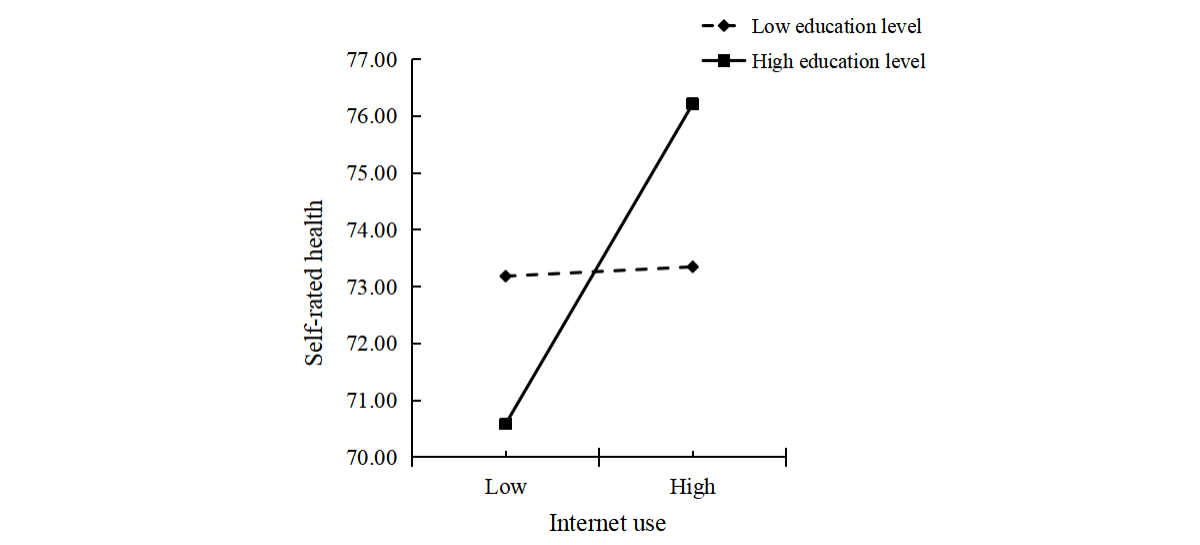
Education level as a moderator in the relationship between internet use and self-rated health.

Similarly, the interaction term between *internet use* and *education level* (B=1.284; *P*=.01) was a predictor of *self-rated health*. Education level positively moderated the relationship between internet use and *self-rated health*, as shown in Figure 5. The results indicated that, at higher education levels, the effect of internet use on self-efficacy was stronger (B=1.752; *P*=.007), whereas at lower education levels, the moderating effect was not statistically significant (B=0.051; *P*=.90), as presented in Table 8. The slope for individuals with higher education was steeper than for those with lower education, indicating that internet use had a greater impact on *self-rated health* among older adults with higher education levels.

Furthermore, this study examined the moderating effect of *education level* on each mediating pathway (Table 9).

**Table 9:** Results of moderated chain mediation analysis.

Indirect effect path and moderating effect			B^a^ (SE; 95% CI)
**Internet use→self-efficacy→self-rated health**			
	Low level of education	0.341 (0.106; 0.151 to 0.573)	
	High level of education	0.627 (0.200; 0.279 to 1.051)	
	Index of moderated mediation	0.216 (0.104; 0.046 to 0.450)	
**Internet use→health literacy→self-rated health**			
	Low level of education	0.745 (0.137; 0.496 to 1.033)	
	High level of education	0.596 (0.151; 0.315 to 0.918)	
	Index of moderated mediation	−0.113 (0.115; −0.347 to 0.106)	
**Internet use→physical exercise→self-rated health**			
	Low level of education	0.304 (0.096; 0.133 to 0.514)	
	High level of education	0.299 (0.109; 0.107 to 0.535)	
	Index of moderated mediation	−0.004 (0.063; −0.135 to 0.117)	
**Internet use→self-efficacy→health literacy→self-rated health**			
	Low level of education	0.180 (0.040; 0.108 to 0.264)	
	High level of education	0.330 (0.074; 0.196 to 0.482)	
	Index of moderated mediation	0.114 (0.047; 0.029 to 0.210)	
**Internet use→self-efficacy→physical exercise→self-rated health**			
	Low level of education	0.012 (0.011; −0.008 to 0.035)	
	High level of education	0.021 (0.020; −0.013 to 0.067)	
	Index of moderated mediation	0.007 (0.008; −0.004 to 0.027)	
**Internet use→health literacy→physical exercise→self-rated health**			
	Low level of education	0.032 (0.015; 0.007 to 0.066)	
	High level of education	0.025 (0.014; 0.005 to 0.058)	
	Index of moderated mediation	−0.005 (0.006; 0.018 to 0.005)	
**Internet use→self-efficacy→health literacy→physical exercise→self-rated health**			
	Low level of education	0.008 (0.004; 0.002 to 0.016)	
	High level of education	0.014 (0.007; 0.003 to 0.031)	
	Index of moderated mediation	0.005 (0.003; 0.001 to 0.012)	

^a^B: unstandardized regression coefficient.

As shown in Table 9, education level significantly moderated the magnitude of the indirect effects along paths 1, 4, and 7 in the relationship between internet use and self-rated health. However, no significant moderating effect was observed in paths 2, 3, 5, and 6. Education level positively moderated the mediating effects of paths 1, 4, and 7 (moderating effects of 0.216, 0.114, and 0.005, respectively), which means that the indirect effects of mediating paths 1, 4, and 7 were stronger at higher education levels and weaker at lower education levels. Overall, hypothesis 9 was supported. Education level may positively moderate the relationship between internet use and self-rated health among older adults.

### Additional Analysis

To ensure the robustness of the findings, we conducted an additional analysis in this study, which aimed to assess the correlation between self-rated health and other measurable indicators and to reduce the subjective reporting bias of self-rated health scores. If the correlation is high, the self-rated health can be considered a more realistic response to the health status of older adults. Due to data availability, the BMI indicator was chosen for analysis in this study. BMI is an important indicator of physical health in older adults, and its recommended range varies according to age. On the basis of the recommendations from the Dietary Guidelines for Chinese Residents and the BMI and Weight Management Guidelines for the Chinese Oldest Old, we set different recommended values for various age groups of older adults. Specifically, the optimal BMI range for older adults aged 60 to 64 years was 18.5-23.9 kg/m^2^; for the age group of 65 to 79 years, the range was 20.0-26.9 kg/m^2^; and for those aged ≥80 years, it was 22.0-26.9 kg/m^2^. Table 10 summarizes the recommended BMI values for specific age groups.

**Table 10:** Age-specific recommended BMI ranges.

Age group (y)	Recommended BMI range (kg/m^2^)
60-64	18.5-23.9
65-79	20.0-26.9
≥80	22.0-26.9

In this study, BMI values were coded as a binary variable. BMI values within the recommended range were coded as 1, and values outside the range were coded as 0. To investigate the association between BMI classification and self-rated health, the Spearman rank correlation test was performed in this study.

The analysis revealed a significant positive correlation (ρ=0.097; *P*=.001) between BMI in the recommended range and better self-rated health. This suggests that older adults with BMI values within the recommended range tend to report better self-rated health. These findings further support the representativeness of self-rated health as an indicator of health status in older adults and justify its use as an explanatory variable in this study.

## Discussion

### Principal Findings

This study provides new insights into the mechanisms linking internet use and health outcomes among older adults, drawing upon media effects theory and the HBM. The findings indicate that internet use enhances self-rated health indirectly through a sequential chain involving self-efficacy, health literacy, and physical exercise. These three factors not only function as independent mediators but also interact cumulatively, suggesting a structured pathway of influence. The results support the view that individual-level psychological and behavioral factors play a critical role in shaping the relationship between internet use and self-rated health among older adults. Furthermore, the mediating effects were more prominent among female participants and those not categorized as low-income. In addition, education level emerged as a significant moderator, affecting the strength of the associations between internet use, self-efficacy, and self-rated health.

### The Association Between Internet Use and Self-Rated Health

The results support hypothesis 1, confirming a significant positive association between internet use and self-rated health among older adults. This finding is consistent with the conclusions drawn by Fjell et al [62] and Wang and Chen [63]. From the perspective of the media effects theory, the internet, as a modern and dynamic media form, facilitates access to diverse platforms such as social media, health-related websites, and e-commerce applications. For older adults, this form of media not only expands access to reliable health information but also enhances opportunities for social connectivity. These functions contribute to improved health perceptions and behaviors. For example, the internet enables timely communication with health care professionals [64], helping older adults overcome common barriers to medical access. As a result, their health conditions are more likely to improve through early intervention and continuous guidance [65]. In addition, the internet increases social interaction among older adults, helping them promote sustained social engagement and promoting their health [66].

### Mediating Roles of Self-Efficacy, Health Literacy, and Physical Exercise

The chain mediating analysis reveals that *self-efficacy*, *health literacy*, and *physical exercise* fully mediate between internet use and the self-rated health of older adults. This finding confirms hypotheses 2, 3, 4, 5, 6, and 8. The result illustrates that the positive impact of internet use on older adults’ self-rated health is entirely manifested through these three mediating factors. Internet access alone does not directly improve health outcomes; it is the additional benefits of self-efficacy, health literacy, and physical exercise during internet use that improve older adults’ self-rated health. These findings align with the media effects theory [16,17], which posits that sustained media engagement can lead to attitudinal and behavioral changes by shaping individuals’ perceptions, knowledge, and actions.

First, internet use is positively associated with self-efficacy. This relationship reflects the media’s role in reinforcing individual agency and perceived control. Consistent with the media effects theory, higher media exposure, such as smartphones, is associated with higher self-efficacy [67]. After engaging with various media platforms, individuals can enhance their self-efficacy through self-motivation and self-regulation [68], as well as by increasing their perception of social support [69]. In addition, the internet offers new learning environments and opportunities through online courses, aiding individuals in gaining confidence and a sense of achievement from learning and mastering new skills, thereby further boosting self-efficacy [70].

Second, higher self-efficacy facilitates the improvement of health literacy among older adults. On one hand, the internet reduces information asymmetry and broadens access to various types of information. It enables older adults to learn about fitness and health knowledge online, significantly improving their health literacy and, consequently, their health levels [20]. On the other hand, within the HBM, self-efficacy plays a crucial role in influencing behavior. It empowers older adults to believe that they can improve their health through a series of actions, encouraging them to actively learn health knowledge, enhance health literacy, and attempt health behavior changes. As self-efficacy increases, older adults become more proactive in engaging in health-related social activities and adopting healthier lifestyles, behaviors that contribute to the enhancement of health literacy [47].

Third, the improvement of health literacy can further enhance the physical exercise of older adults, thereby promoting overall health improvements. Existing research confirms the positive role of the internet in enhancing physical exercise. Older adults can watch sports broadcasts, share exercise experiences, and participate in online sports communities, gaining a wealth of sports-related information and knowledge [71]. This digital exposure supports exercise planning [72] and increases awareness of the benefits of regular activity [73], encouraging consistent participation in physical exercise. Moreover, advancements in cognitive capacity enable behavioral change. Enhanced health literacy strengthens the ability to seek and understand health information, facilitating the decision-making process for engaging in physical exercise [74].

However, the expected pathway from self-efficacy to physical exercise (hypothesis 6) was not supported. One possible explanation is that physical exercise is influenced not only by internal motivation (eg, self-efficacy) but also by external factors such as social support [75]. Previous studies have shown that social support plays a critical role in shaping older adults’ willingness to engage in physical activity [76,77]. In addition, there is an interaction between self-efficacy and social support, and older adults’ participation in physical exercise increases only when both levels are high [78]. Compared with older adults in Western countries who regularly engage in individual exercise, older adults in East Asian societies are more likely to participate in group physical activities, such as square dancing [79], which are often more dependent on social support for motivation. Thus, the effect of self-efficacy on physical activity behavior may be weakened or masked in contexts where social support is lacking. Another possible explanation is that physical decline in older adults may diminish the predictive power of psychological constructs such as self-efficacy. For example, in a study by Liu et al [80], 41% of older adult respondents reported that their physical exercise was constrained by physical limitations. As physical functioning deteriorates, actual physical capability may replace self-efficacy as a more dominant determinant of physical exercise.

### Differences in Mediation Effects Among Different Subgroups

Heterogeneity analyses revealed significant differences in mediation effects across subgroups. The stronger mediation effect observed among female participants may reflect sex differences in technology use patterns and health behavior responsiveness. Previous research suggests that female individuals tend to be more proactive in seeking health information and are more likely to use this information to promote their health [8,81].

Regarding income, findings suggest that older adults with higher per capita household incomes are more likely to improve their self-rated health through internet use. This may be because higher-income groups tend to have greater digital access, higher levels of education, and better health literacy [82-84]. As a result, they can use internet resources more effectively to improve their self-efficacy and physical exercise through internet use, thus enhancing their health.

In contrast, differences in mediating effects between urban and rural areas were relatively small. This suggests that the mechanisms that translate into better self-rated health through internet use were similar across residential settings. This finding provides promising evidence that digital health interventions have the potential to close the urban-rural health gap if internet access and training are ensured [85].

### Moderating Role of Education Level

The results partially support hypothesis 9. Education level significantly moderates the relationship between internet use and both self-efficacy and self-rated health. Education level can influence literacy, cognitive levels, flexibility in searching for information, and technology acceptance [86], enabling older adults with higher education to more readily grasp internet-related knowledge and skills, thereby enhancing their self-efficacy. They are more likely to find suitable health resources and support services online, helping to improve their health.

Moderated mediation analysis further revealed that education strengthens three indirect pathways from internet use through (1) self-efficacy alone; (2) self-efficacy and health literacy; and (3) the full chain, including self-efficacy, health literacy, and physical exercise. The consistent presence of self-efficacy in all moderated pathways suggests that it is the key point at which education exerts its influence. This finding is in line with and further supports a previous study [11], which reported that those with higher education often have greater autonomy and control, indicating a strong and stable self-efficacy.

However, the education level did not moderate the effects of internet use on health literacy or physical exercise directly. According to the HBM, health literacy in this context may arise more from perceived usefulness and motivation to learn than from formal educational attainment. Similarly, participation in physical exercise among older adults may be influenced more by physical condition, environment, or routine than by educational background. In both cases, the effect of education may be indirectly working through self-efficacy rather than directly.

### Policy Suggestions

On the basis of the findings, this study offers targeted recommendations to promote older adults’ health in the digital age from both societal and individual perspectives.

At the societal level, efforts should focus on building age-friendly digital environments. Governments can support the development of internet platforms tailored to older adults, especially in areas such as health information, telemedicine, and physical exercise. Community-based digital training programs should be expanded to improve not only technical skills but also confidence in internet use. Health institutions and family physician teams should strengthen health education by combining offline methods (eg, lectures) with digital formats (eg, short videos and official accounts), improving access to health information across education levels. In addition, it is suggested to use mobile apps to establish online exercise communities at the community level, creating an older adult–friendly and supportive social environment, thereby motivating older adults to engage more actively in physical exercise.

At the individual level, enhancing self-efficacy is key. Older adults should be encouraged to engage in structured social activities such as volunteering or digital learning, which reinforce confidence through task achievement and social support.

### Limitations and Future Research

This study has several limitations. First, the use of cross-sectional data restricts the ability to infer causal relationships between internet use and health outcomes. Second, the outcome variable was based on self-rated health, introducing the potential for reporting biases. Third, the measurement of internet use was limited to frequency, without accounting for use types, which constrains the interpretation of how specific internet behaviors relate to health. Fourth, although key demographic and health-related variables were controlled, unmeasured confounders such as cognitive functioning, personality traits, and digital literacy may still affect the results. In addition, converting the health literacy scale into categorical levels may have reduced data sensitivity.

Future research should address these limitations by using longitudinal designs, incorporating multidimensional measures of internet engagement, using objective physiological health indicators, and controlling for a broader range of confounding variables. Furthermore, modeling health literacy as a continuous construct rather than converting it into discrete categories may help preserve its full variability and improve the sensitivity of mediation analyses.

### Conclusions

The study indicates that internet use can impact the health of older adults through individual and chain mediating effects of self-efficacy, health literacy, and physical exercise, with education level moderating several mediating paths. The research holds theoretical and practical value for exploring positive aging from the perspective of internet use. First, it offers a new research perspective for proactive aging and the construction of digital older adult care systems. Second, most previous studies have only discussed individual mediating effects, neglecting the chain impact of multiple mediators. Moreover, the study considers the moderating role of education level on mediating paths, providing a new perspective for understanding the influence of cultural capital on health. Finally, this study proposes a few suggestions to support healthy aging, including promoting age-friendly digital platforms, expanding digital training and health education, and encouraging older adults to engage in volunteering or digital learning to enhance self-efficacy.

## Appendix

Multimedia Appendix 1 Selected questionnaire questions from the Psychology and Behavior Investigation of Chinese Residents 2021.

Multimedia Appendix 2 Detailed information on survey regions.

## Abbreviations

HBM: health belief model
PBICR: Psychology and Behavior Investigation of Chinese Residents

## Data Availability

The Psychology and Behavior Investigation of Chinese Residents (PBICR) is an open access, large-sample, nationwide representative study. It regularly collects high-quality microdata from representative Chinese populations, including the general population, special groups, and patients with specific diseases, to analyze psychological and behavioral issues among Chinese residents. The study currently provides access to data from 2020 and 2021, which can be requested by following the detailed procedure outlined on the PBICR website [87]. On the website, applicants may click the section “Open Database” to view the full data access instructions, including contact information and required documents.
