# Supplementary material for: The Chain Mediation Role of Self-Efficacy, Health Literacy, and Physical Exercise in the Relationship Between Internet Use and Older Adults’ Health: Cross-Sectional Questionnaire Study
Source: J Med Internet Res. 2025 Jul 1;27:e73242. doi: 10.2196/73242 (PMC12264443; doi:10.2196/73242)
Supplement: Multimedia Appendix 1 [file jmir_v27i1e73242_app1.doc]

**Multimedia Appendix 1**

**Selected Questionnaire Questions from PBICR 2021**

We would like to invite you to participate in an anonymous online survey, which will take approximately 20-30 minutes. This informed consent form provides you with information to help you decide whether to participate in this survey. Please read it carefully, and if you have any questions, feel free to ask the researchers.

1. If you agree to participate in our survey, please click on Agree below and start the questionnaire. [Single Choice] *

○Yes

○No (Please skip to the end of Questionnaire No. to submit your answer.)

2.Your gender: [Single Choice] *

○Male

○Female

3. Your age range (years): [Single Choice] *

| ○≤18 | ○19-25 | ○26-30 | ○31-35 | ○36-40 |
| --- | --- | --- | --- | --- |
| ○41-45 | ○46-50 | ○51-55 | ○56-59 | ○60-65 |
| ○66-70 | ○71-75 | ○76-80 | ○81-85 | ○86-90 |
| ○91-95 | ○96-100 | ○≥101 |  |  |

4. Your usual residence: [Single Choice] *

○Urban

○Rural

5. Your per capita monthly household income (yuan): [Single Choice] *

| ○≤1500 | ○1501-3000 | ○3001-4500 | ○4501-6000 |
| --- | --- | --- | --- |
| ○6001-7500 | ○7501-9000 | ○9001 -○10500 | ○10501-12000 |
| ○12001-13500 | ○13501-15000 | ○≥15001 |  |

6. Your nationality: [Single Choice]*

○ Han ethnicity ○ Ethnic minority _________________*

7. Your political affiliation: [Single Choice]*

○ Party member

○ Party probationary member

○ Communist Youth League member

○ Member of the Revolutionary Committee Of The Chinese Kuomintang

○ Member of the China Democratic League Central Committee

○ Member of the China National Democratic Construction Association

○ Member of the China Association for the Promotion of Democracy

○ Member of the Chinese Peasants and Workers Democratic Party

○ Member of the China Zhi Gong Party

○ Member of the Jiusan Society

○ Member of the Taiwan Democratic Self-Government League

○ Non-affiliated Person

○ Masses

8. Your highest level of education: [Single Choice]*

| ○ No formal education | ○ Primary school | ○ Junior high school |
| --- | --- | --- |
| ○ Technical secondary school | ○ Senior high school | ○ Associate degree |
| ○ Bachelor's degree | ○ Master's degree | ○ Doctoral degree |

9. Your marital status: [Single Choice] *

○Unmarried

○Married (including first marriage with a spouse, remarriage with a spouse, remarriage with a spouse)

○Divorced

○Widowed

10. People who have lived with you in the past two months: [Multiple Choice]*

□ Living alone

□ Spouse

□ My own father

□ My own mother

□ Spouse’s father

□ Spouse’s mother

□ Brothers and sisters (if any, please fill in the number of people)_________________*

□ Son (if any, please fill in how many people)_________________*

□ Daughter (if any, please fill in how many people)_________________*

□ Nanny (if available, please fill in how many people)_________________*

□ Dormitory roommates (if any, please fill in how many people)_________________*

□ Other (if any, please fill in how many people)_________________*

11. Your current type of medical insurance: [Single Choice]*

○ Urban and rural resident medical insurance

○ Urban employee medical insurance

○ Commercial medical insurance

○ Public medical insurance

○ Out-of-pocket Spending

12. Have you currently been diagnosed with the following diseases? [Multiple Choice]*

□ None

□ Hypertension

□ Stroke (cerebral infarction, cerebral hemorrhage)

□ Coronary heart disease

□ Dyslipidemia

□ Diabetes

□ Malignant tumor

□ Asthma

□ Chronic obstructive pulmonary disease (COPD)

□ Chronic kidney disease

□ Chronic enteritis

□ Chronic gastritis

□ Viral hepatitis (such as hepatitis B)

□ Fatty liver disease

□ Alzheimer's disease (senile dementia)

□ Parkinson's disease

□ Emotional disorders (anxiety, depression, etc.)

□ Other_________________*

13. Your height (unit: cm) [fill in the blank question]*

(Just fill in the number, such as 172)

_________________________________

14. Your weight (in pounds) [fill in the blank question]*

(Just fill in the number, such as 120)

_________________________________

15.How would you rate your health today?

This scale has numbers from 0 to 100, with 100 representing the best health condition you can imagine; 0 represents the worst health condition you can imagine. Please indicate your current health condition and pull the slider to the corresponding number.

[Enter a number from 0 (the worst health condition you imagine) to 100 (the best health condition you imagine)]*

________________________________

16. Please select the most suitable option for you: [Matrix Single Choice]*

|  | Strongly disagree | Disagree | Neither agree nor disagree | Agree | Strongly agree |
| --- | --- | --- | --- | --- | --- |
| I will be able to achieve most of the goals I have set for myself | ○ | ○ | ○ | ○ | ○ |
| When faced with difficult tasks, I am confident that I can complete them | ○ | ○ | ○ | ○ | ○ |
| Overall, I believe I can achieve results that are important to me | ○ | ○ | ○ | ○ | ○ |
| I believe that as long as I make up my mind, I can succeed with any effort | ○ | ○ | ○ | ○ | ○ |
| I will be able to successfully overcome many challenges | ○ | ○ | ○ | ○ | ○ |
| I am confident that I can effectively complete many different tasks | ○ | ○ | ○ | ○ | ○ |
| Compared to others, I can complete most tasks very well | ○ | ○ | ○ | ○ | ○ |
| Even if things are difficult, I can still perform well | ○ | ○ | ○ | ○ | ○ |

17. How often do you typically use the following media? [Matrix Single Choice]*

|  | Never used | Occasionally (≤1 day/week) | Sometimes used (2-3 days/week) | Frequently used (4-5 days/week) | Almost every day (6-7 days/week) |
| --- | --- | --- | --- | --- | --- |
| Newspaper | ○ | ○ | ○ | ○ | ○ |
| Magazine | ○ | ○ | ○ | ○ | ○ |
| Broadcast | ○ | ○ | ○ | ○ | ○ |
| Television | ○ | ○ | ○ | ○ | ○ |
| Books (non textbooks) | ○ | ○ | ○ | ○ | ○ |
| Personal computers (including tablets) | ○ | ○ | ○ | ○ | ○ |
| Smartphone | ○ | ○ | ○ | ○ | ○ |

18. From very easy to very difficult, please rate the difficulty of the following behaviors for you: [Matrix Single Choice]*

|  | Very difficult | Difficult | Easy | Very easy |
| --- | --- | --- | --- | --- |
| Find treatment information for your illness | ○ | ○ | ○ | ○ |
| Understand the instructions accompanying the medication you are taking | ○ | ○ | ○ | ○ |
| Assessing the advantages and disadvantages of different treatment options | ○ | ○ | ○ | ○ |
| Call an ambulance in an emergency situation | ○ | ○ | ○ | ○ |
| Find information on how to deal with mental health issues such as stress or depression | ○ | ○ | ○ | ○ |
| Understand why one needs to undergo health check ups (such as breast examination, blood glucose measurement, blood pressure measurement) | ○ | ○ | ○ | ○ |
| Determine the type of vaccine you may need to receive | ○ | ○ | ○ | ○ |
| Take appropriate measures to prevent diseases based on the advice of family and friends | ○ | ○ | ○ | ○ |
| Discover activities that are beneficial for your mental health, such as meditation, exercise, walking, Pilates, etc | ○ | ○ | ○ | ○ |
| Understand the information on how to become healthier in the media (such as the Internet, newspapers, magazines) | ○ | ○ | ○ | ○ |
| Identify which daily behaviors (such as drinking and eating habits, exercise, etc.) will have an impact on your health | ○ | ○ | ○ | ○ |
| If interested, participate in a sports organization or fitness class | ○ | ○ | ○ | ○ |

19.How much time have you spent on the following activities in the past week? [Matrix Single Choice]*

|  | Not done | <30 minutes/week | 30-59 minutes/week | 1-3 hours/week | >3 hours/week |
| --- | --- | --- | --- | --- | --- |
| Fitness exercises (such as consecutive movements, lifting dumbbells, etc.) | ○ | ○ | ○ | ○ | ○ |
| Walking | ○ | ○ | ○ | ○ | ○ |
| Swimming | ○ | ○ | ○ | ○ | ○ |
| Cycling | ○ | ○ | ○ | ○ | ○ |
| Exercise with sports equipment (such as treadmills, trampolines, etc.) | ○ | ○ | ○ | ○ | ○ |
| Other aerobic exercises (such as running, playing table tennis, etc.) | ○ | ○ | ○ | ○ | ○ |
