# Supplementary material for: The Chain Mediation Role of Self-Efficacy, Health Literacy, and Physical Exercise in the Relationship Between Internet Use and Older Adults’ Health: Cross-Sectional Questionnaire Study
Source: J Med Internet Res. 2025 Jul 1;27:e73242. doi: 10.2196/73242 (PMC12264443; doi:10.2196/73242)
Supplement: Multimedia Appendix 2 [file jmir_v27i1e73242_app2.doc]

**Multimedia Appendix 2**

**Detailed Information of Survey Regions**

**23 Provinces:**

Hebei Province (6 cities): Shijiazhuang, Tangshan, Handan, Baoding, Cangzhou, Hengshui

Shanxi Province (6 cities): Taiyuan, Datong, Shuozhou, Lüliang, Jinzhong, Linfen

Liaoning Province (3 cities): Shenyang, Dalian, Jinzhou

Jilin Province (3 cities): Changchun, Yanbian Korean Autonomous Prefecture, Siping

Heilongjiang Province (3 cities): Harbin, Suihua, Qiqihar

Jiangsu Province (3 cities): Nanjing, Suzhou, Nantong

Zhejiang Province (7 cities): Hangzhou, Ningbo, Wenzhou, Shaoxing, Jiaxing, Quzhou, Taizhou

Anhui Province (7 cities): Hefei, Wuhu, Bengbu, Fuyang, Chuzhou, Lu’an, Bozhou

Fujian Province (4 cities): Fuzhou, Xiamen, Zhangzhou, Sanming

Jiangxi Province (4 cities): Nanchang, Shangrao, Ganzhou, Jingdezhen

Shandong Province (7 cities): Jinan, Qingdao, Yantai, Weifang, Tai’an, Weihai, Linyi

Henan Province (7 cities): Zhengzhou, Anyang, Hebi, Puyang, Shangqiu, Zhoukou, Nanyang

Hubei Province (5 cities/prefectures): Wuhan, Yichang, Xiangyang, Huanggang, Enshi Tujia and Miao Autonomous Prefecture

Hunan Province (4 cities): Changsha, Hengyang, Changde, Yiyang

Guangdong Province (4 cities): Guangzhou, Shenzhen, Jiangmen, Dongguan

Hainan Province (3 cities): Haikou, Qionghai, Wenchang

Sichuan Province (5 cities): Chengdu, Luzhou, Mianyang, Guangyuan, Bazhong

Guizhou Province (4 cities): Guiyang, Zunyi, Liupanshui, Tongren

Yunnan Province (4 cities/prefectures): Kunming, Pu’er, Dehong Dai and Jingpo Autonomous Prefecture, Chuxiong Yi Autonomous Prefecture

Shaanxi Province (4 cities): Xi’an, Xianyang, Weinan, Hanzhong

Gansu Province (3 cities): Lanzhou, Wuwei, Zhangye

Qinghai Province (3 cities/prefectures): Xining, Haixi Mongol and Tibetan Autonomous Prefecture, Haidong

**5 Autonomous Regions:**

Inner Mongolia Autonomous Region (3 cities): Hohhot, Tongliao, Bayannur

Guangxi Zhuang Autonomous Region (3 cities): Nanning, Guilin, Guigang

Tibet Autonomous Region (3 cities): Lhasa, Nagqu, Chamdo

Ningxia Hui Autonomous Region (4 cities): Yinchuan, Wuzhong, Guyuan, Zhongwei

Xinjiang Uygur Autonomous Region (4 cities/prefectures): Ürümqi, Hami, Changji Hui Autonomous Prefecture, Ili Kazakh Autonomous Prefecture

**4 Municipalities Directly Under the Central Government:**

Beijing, Tianjin, Shanghai, Chongqing

**Note:** The recruitment areas for this survey do not include the Hong Kong Special Administrative Region, Macao Special Administrative Region, or Taiwan Province.
